# Distribution of *Phoxinus eos*, *Phoxinus neogaeus*, and Their Asexually-Reproducing Hybrids (Pisces: Cyprinidae) in Algonquin Provincial Park, Ontario

**DOI:** 10.1371/journal.pone.0013185

**Published:** 2010-10-04

**Authors:** Jonathan A. Mee, Locke Rowe

**Affiliations:** 1 Department of Zoology, University of British Columbia, Vancouver, British Columbia, Canada; 2 Department of Ecology and Evolutionary Biology, University of Toronto, Toronto, Ontario, Canada; Montreal Botanical Garden, Canada

## Abstract

Hybrid *Phoxinus* are one of the few asexually reproducing vertebrates species. The distribution of hybrid *Phoxinus* among lakes in Algonquin Park, Ontario, was evaluated relative to the distribution of parental species and relative to physiochemical lake characteristics. No association between the distribution of hybrids and the distribution of parental species was found, suggesting that the hybrids can successfully coexist with either parental species. In addition, we found no association between hybrid distribution and the physiochemical characteristics of lakes, suggesting that the hybrids are generalists with respect to the ecological niches available in Algonquin Park. There was a difference between the physiochemical characteristics of lakes with and without the parental species *P. neogaeus*. The lakes containing *P. neogaeus* were lower elevation than the lakes containing the other parental species, *P. eos*. The difference in distribution between the parental species may therefore be due to different dispersal abilities, to later arrival following post-glacial dispersal, or to differences in ecology. These results suggest that asexual reproduction is a successful strategy for hybrid *Phoxinus* in Algonquin Park because these sperm-dependent asexual hybrids are able to survive and persist regardless of which parental species is present, and regardless of the physiochemical characteristics of their habitat.

## Introduction

The evolution of sex and maintenance of sex are important unanswered questions in evolutionary biology [1]. Although vertebrates are ancestrally sexual, asexuality has arisen via hybridization at least 90 times in the vertebrate lineage [2], [3]. Asexual vertebrates provide an opportunity to evaluate the reasons why asexual reproduction is rare among vertebrates, and more generally how asexual and sexual species are able to coexist. All known asexual fishes and amphibians, which together comprise the majority of known asexual vertebrates, are sperm-dependent. The coexistence of sperm-dependent asexuals with their sexually reproducing sperm donors is particularly problematic because the competitive success of the hybrids, resulting from the two-fold “cost of sex” [4], [5], [6], [7], [8], will ultimately lead to the extinction of both hybrids and parentals. Asexuality may thus be rare among vertebrates because asexuals arise very rarely, or, once arisen, asexuals may be unable to persist. There are a variety of factors that might contribute to the successful coexistence of asexually- and sexually-reproducing species [9], [10], [11], [12]. One such factor is the existence of important ecological differences between the asexuals and sexuals. If these ecological differences are important in allowing the persistence of the asexuals, then one would expect that coexistence would be successful in some habitats (e.g., lakes with particular physiochemical characteristics) and not in others. The broad-scale distribution of asexuals may therefore be correlated with broad-scale habitat variation.

Hybrid fish in the genus *Phoxinus* have originated from hybridization between *P. eos* Cope, 1862, and *P. neogaeus* Cope, 1867 [13]. The most striking consequence of hybridization in the case hybrid *Phoxinus* is that the hybrids are all female and reproduce via gynogenesis – a mode of sperm-dependent asexual reproduction in which sperm from a parental species is required to stimulate egg development, but not for its genetic contribution [10]. A consequence of this sperm-dependent asexual reproduction is that the hybrids must always coexist with at least one parental species. The hybrid *Phoxinus* populations studied to date usually coexist with only *P. eos*, although populations coexisting with *P. neogaeus* or with both parental species are not uncommon [14], [15], [16], [17]. The two parental species are both widespread in North America and do sometimes coexist, but there is no evidence of ongoing or even recent hybridization between *P. eos* and *P. neogaeus* [10], [14], [17], [18], [19]. Angers & Schlosser [14] estimated that hybridization between *P. eos* and *P. neogaeus* occurred during the last glaciation, roughly 50 000 years ago, when all North American freshwater fish species were confined to a few glacial refugia. It is possible that many hybrid clones originated at the time when hybrids were formed, but only certain clones (or lineages) were able to survive and expand their range along with the parental species following de-glaciation. The hybrid *Phoxinus* “species”, which, in reality, consists of a number of clonal lineages that originated at the time of hybridization [14], is thought to be widely distributed in Canada and the northern United States.

Given that hybrid *Phoxinus* are asexual, and in many hybrid populations most of the individuals are genetically identical [14], [17], it is conceivable that a lack of genetic diversity limits the hybrids to a subset of the lakes in which the parental species are able to survive. Alternatively, the hybrids might be generalists and can coexist with the parentals regardless of a given lake's physical or chemical characteristics. Also, it is important that the hybrid clones are able to solicit sperm from a parental species male. It may be that a given hybrid clone can solicit sperm from only one parental species. Alternatively, a single clone may be able to solicit sperm from, and hence coexist with, either parental species. There is evidence that the hybrids do have a “general purpose” clonal genotype with respect to their ecological niche [17], and there may be important ecological differences between the hybrids and parental species [17].

The existence of hybrid *Phoxinus* was not widely known until recently [13], and many existing sampling records of *Phoxinus* do not mention any hybrids. Many museum collections of *P. eos* and *P. neogaeus* are actually misidentified hybrids (J.A.M. personal observations of collections from the University of British Columbia Fish Museum and the New Brunswick Museum). As such, we have little information about the existence of hybrids in lakes over most of the range of *P. eos* and *P. neogaeus*. An inventory of fish species in Algonquin Park, Ontario, completed in 1991 did include hybrid *Phoxinus*, and this inventory provides an opportunity to study the determinants of the distribution of hybrid *Phoxinus* over a broad range (147 lakes containing *Phoxinus* over more than 7700 km^2^). In the present study, we compare the distribution of hybrids to that of parental *Phoxinus* and relative to physical and chemical characteristics of lakes in Algonquin Park, Ontario. This comparison of the distributions of hybrid and parental *Phoxinus* was intended to address the hypothesis that, over the range of lakes and habitats sampled in Algonquin Park, hybrids and parental species should be constrained to particular, and perhaps different, physiochemical environments.

## Materials and Methods

Data on the distribution of hybrid *Phoxinus* and parental species in Algonquin Park were obtained from a database of collections housed at the Royal Ontario Museum (ROM). Algonquin Park covers an area of 7725 km^2^ and encompasses the headwaters of five major watersheds flowing into both the Ottawa River and the Great Lakes. The ROM collection was compiled from an inventory of fish species in Algonquin Park from 1989 to 1991. Sampling for the Algonquin Park inventory was conducted between May and August. A single voucher specimen of each species (including the hybrid “species” and both parental *Phoxinus* species) found in each lake was retained for the ROM collection. *Phoxinus eos* were identified by the presence of a narrow, sharply-defined lateral stripe, a distinct supralateral stripe that is often solid at the anterior end and broken at the posterior end, a small, narrow, upturned mouth (usually not reaching beyond the anterior margin of the eye), and a highly coiled, narrow gut [13], [16], [20]. *Phoxinus neogaeus* were identified by the presence of a broad, diffuse lateral stripe, the absence of a supralateral stripe, a large, wide, horizontal mouth (often reaching beyond the middle of the eye), and a simple S-shaped, broad gut [13], [16], [20]. Hybrids were differentiated from parental *Phoxinus* species by the presence of an intermittent second lateral stripe, intermediate mouth size, and intermediate gut coiling [13], [16], [20]. We note that, in other studies, *Phoxinus* with intermediate morphology have been positively identified as hybrids using the genetic techniques of Binet and Angers [19]. There is, however, no guarantee that the characters used would have always accurately differentiated hybrid and parental *Phoxinus*. Nonetheless, there is no expectation that misidentification would be biased in any way. The collectors were aware of the presence of hybrids in these lakes and would have been just as likely to mistake a hybrid for a parental as *vice versa*.

**Figure 1:**
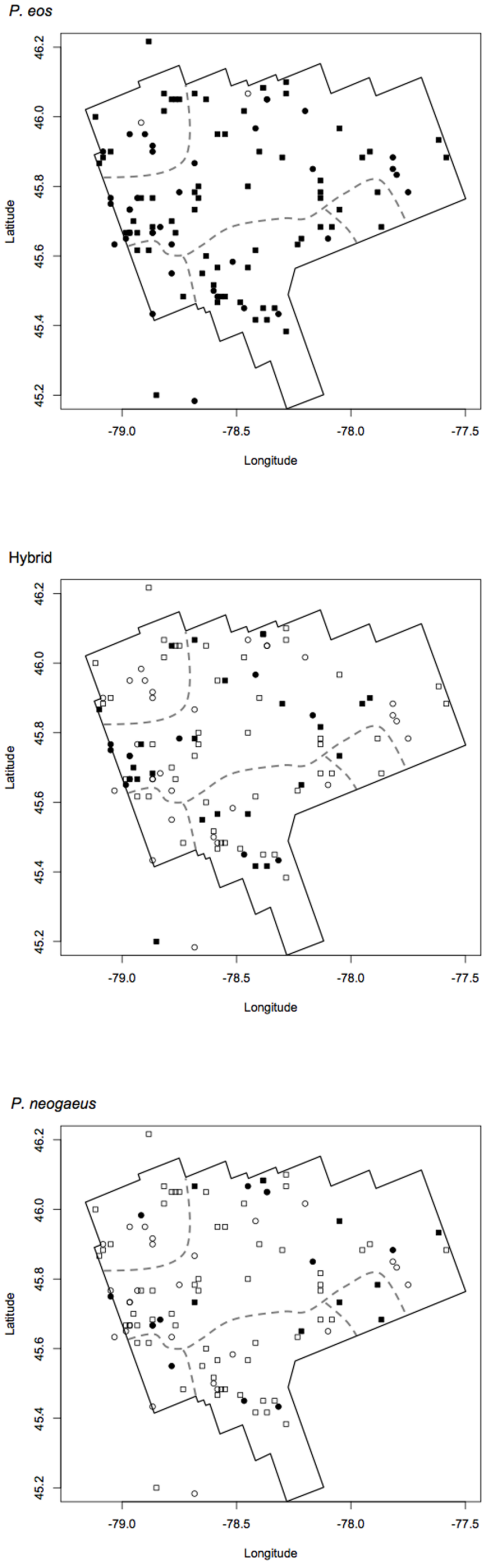
The distribution of *Phoxinus eos*, *P. neogaeus*, and hybrids among lakes sampled for an inventory of fish species in Algonquin Park Ontario. Each symbol represents one lake that contained at least one of *P. eos*, *P. neogaeus*, and hybrids. Solid symbols represent presences and open circles represent absences. Square symbols represent lakes used in the analysis of lake physiochemical characteristics, while circles represent lakes that were excluded form the analysis due to missing physiochemical data. For scale, each 0.2 degrees of latitude equals approximately 22.2 km. Thirty-nine lakes for which latitude and longitude were not available are not represented in this figure. Gray dashed lines represent approximate boundaries of major watersheds within Algonquin Park.

There are 147 lakes in the ROM database that contained at least one of *P. eos*, *P. neogaeus*, or hybrids (figure 1). Although the ROM database represents neither a comprehensive nor a random sample of Algonquin Park lakes, there is no reason to think that the sampling should bias the presence or absence of hybrids or parental species among lakes in the database. Sampling gear varied between lakes, but not in a systematic way, and detectability of hybrid and parental *Phoxinus* should not differ with different sampling gear – when *Phoxinus* species do coexist in a lake, they shoal together and are usually caught together. *Phoxinus* catch rate at a given site within a given lake does not appear to vary seasonally from May through November (JAM, personal observation), and there is good evidence that *Phoxinus* do not stray much from a given site within a lake, even from year to year [21]. We note that there must have been some lakes where the collectors failed to catch particular species or failed to keep voucher specimens representative of all the species caught in a given lake. The presence of error in the dataset, however, does not preclude an analysis using this dataset. We had no a priori reason to think that the degree of error in the ROM dataset would prevent us from detecting any real patterns based on the presence or absence of these species. Fisher's exact test was used to determine whether the presences of hybrids and parental species were correlated.

There were four lakes in the ROM dataset where only hybrids were found. Since hybrid reproduction requires the involvement of a parental male, the absence of both parental species must reflect the collectors' inability to capture a parental species in these four lakes. We use this to calculate a rough estimate of the rate of false absences in the dataset. The ratio of lakes with *P. eos* (129 lakes) to those with *P. neogaeus* (23 lakes) was 5.6∶1, and this ratio ought not to have been affected by false absences in the dataset unless the rate of false absences was not equal among *Phoxinus* species (which is unlikely). The closest approximation of this 5.6∶1 ratio of *P. eos* to *P. neogaeus*, with at least one parental species added to each of the four lakes without any parental species, is achieved if we assume all four of the lakes contained *P. eos* and one of the lakes contained *P. neogaeus*. This brings the total number of hybrid-containing lakes also expected to contain *P. eos* and *P. neogaeus* to 45 and 10, respectively. Since *P. eos* was absent from 4 of the 45 lakes with hybrids where it was expected to occur (8.9% rate of false absences), and *P. neogaeus* was absent from 1 of the 10 lakes with hybrids where it was expected to occur (10% rate of false absences), the rate of false absences in the dataset was estimated to be, on average, 9.4%.

Data on physical and chemical characteristics of the sampled lakes in the ROM survey were obtained from the Ontario Ministry of Natural Resources (MNR) and the Ministry of the Environment (MOE). Eight variables were taken from the MNR and MOE data sets: surface area, maximum depth, elevation, pH, conductivity, dissolved organic carbon concentration, calcium concentration, and SO_4_ concentration. The pH, conductivity, and chemical concentration data were collected in surveys conducted from June to November (mostly October and November) from 1981 and 1992. Data for other physical variables were taken from a variety of surveys conducted from 1936 to 1992. It is important to note that the years over which the fish sampling for the ROM database was conducted coincide quite closely with years when the water chemistry variables were measured. Although the water chemistry data are based on a measurement at a single time point and do not reflect seasonal variation, they are, nonetheless, useful for identifying general differences in water chemistry between lakes [22]. It is unlikely that any anthropogenic disturbance would have affected the water chemistry of the lakes in Algonquin Park between the time of water chemistry sampling and the ROM fish inventory. Ever since the early 1970's, strict forestry management guidelines have required that hundreds of meters of buffer be left between forestry operations and any lake or mapped stream (M. S. Ridgway, personal communication, 2010). Some of the data for surface area, maximum depth, and elevation were collected many years prior to the fish sampling, but these characteristics are unlikely to have changed in the intervening years. In total, 70 lakes had no missing physiochemical data and were used in the analysis. Of these 70 lakes, 24 contained hybrids, 9 contained *P. neogaeus*, and all contained *P. eos*. So, while we were able to evaluate the physiochemical characteristics associated with hybrid and *P. neogaeus* presence or absence, we were unable to evaluate the characteristics associated with *P. eos* presence or absence due to a lack of *P. eos* absences.

A discriminant function analysis (DFA) conducted using the R programming language [23] was used to determine whether either *P. neogaeus* or hybrid *Phoxinus* tend to be restricted to a subset of lakes with particular physical or chemical characteristics. A DFA essentially uses the input variables (i.e., the physiochemical variables) to construct a model that predicts the membership of each lake into one of two groups (e.g., lakes where a particular species was present or absent). Since unequal group sizes will lead to an overestimation of the ability of the DFA model to predict which lakes belong in which group (because lakes from the largest group are likely to be classified into the largest group by chance alone), Cohen's kappa statistic (K) [24], [25], [26] was used to evaluate how well the DFA model performed in predicting group membership. Cohen's kappa gives the chance-corrected proportion of lakes correctly classified by the DFA model. The probability that K is significantly greater than zero was evaluated using a Z statistic. Wilks' lambda was then used as a test statistic to evaluate whether differences in physiochemical characteristics were associated with species presence or absence.

## Results

Hybrid *Phoxinus* were found in 9 of the 27 lakes with both *P. eos* and *P. neogaeus*, none of the 5 lakes with only *P. neogaeus*, and 32 of the 111 lakes with only *P. eos*. The estimated rate of false absences in the dataset (9.4%) predicts 14 false *P. eos* absences, 3 false *P. neogaeus* absences, and 4 false hybrid absences. It is possible to correct for these false absences in such a way that maximizes either a negative or positive correlation between the presences of species. There was no significant correlation between the presences of hybrids and *P. eos* (table 1). Accounting for the potential false absences in the dataset allows the possibility that the presences of hybrids and *P. eos* were either positively or negatively correlated (maximum negative correlation: odds ratio  = 0, p<0.001; maximum positive correlation: odds ratio  =  infinite, p<0.001). There was also no significant correlation between the presences of hybrids and *P. neogaeus*, regardless of how the false absences are accounted for (maximum negative correlation: odds ratio  = 0.63, p = 0.3104; maximum positive correlation: odds ratio  = 2.01, p = 0.0999). There was a negative correlation between the presences of *P. eos* and *P. neogaeus* (odds ratio  = 0.20, p = 0.0238), and accounting for false absences allows only the possibility of a negative correlation (maximum negative correlation: odds ratio  = 0, p<0.001; maximum positive correlation: odds ratio  =  infinite, p = 0.5728).

**Table 1:** Results of Fisher's exact tests of correlation among presences of hybrid and parental *Phoxinus* species in Algonquin Park lakes.

	Maximum negative correlation^1^		Observed correlation		Maximum positive correlation^1^	
Species	Odds ratio (95% conf. int.)	p	Odds ratio (95% conf. int.)	p	Odds ratio (95% conf. int.)	p
*P. eos* & hybrids	**0** (0 to 0.07)	**<0.001**	0.53 (0.11 to 2.82)	0.4566	**infinite** (10.33 to infinite)	**<0.001**
*P. neogaeus* & hybrids	0.63 (0.23 to 1.55)	0.3104	0.86 (0.32 to 2.17)	0.8301	2.01 (0.85 to 4.70)	0.0999
*P. eos* & *P. neogaeus*	**0** (0 to 0.15)	**<0.001**	**0.20** (0.04 to 0.99)	**0.0238**	infinite (0.20 to infinite)	0.5728

1 Maximum negative and positive correlations between species are based on accounting for potential false absences in the dataset in such a way that maximizes either negative or positive correlations.

**Figure 2:**
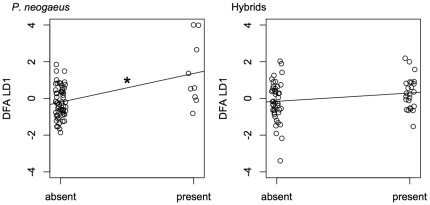
Results of discriminant function analyses (DFA) of the abiotic lake characteristics associated with the presence of *P. neogaeus* and hybrid *Phoxinus* in Algonquin Park lakes. A small amount of random horizontal scatter has been added to the points so overlapping points can be distinguished more easily. Higher DFA LD1 scores for *P. neogaeus* correspond to lakes that are larger, shallower, and lower elevation, and have higher pH, lower conductivity, higher dissolved organic carbon content, lower calcium concentration, and higher SO_4_ concentration. Higher DFA LD1 scores for hybrids correspond to lakes that are smaller, deeper, and lower elevation, and have higher pH, lower conductivity, higher dissolved organic carbon content, lower calcium concentration, and lower SO_4_ concentration. An asterisk indicates a significant correlation between species presence and the abiotic lake characteristics. Lines illustrate the difference in mean LD1 score between groups.

There was a significant association between physiochemical characteristics and the presence of *P. neogaeus* (DFA: df  = 68, Wilks' lambda  = 0.7811, p = 0.04551)(figure 2), and the DFA model reliably predicted group membership despite different group sizes (df  = 68, K = 0.4656, Z = 1.702, p = 0.0446). Lakes containing *P. neogaeus* tended to be larger and shallower, and have a lower elevation, higher pH, greater conductivity, higher dissolved organic carbon content, higher calcium concentration, and higher SO_4_ concentration (table 2). In contrast, there was no association between physiochemical lake characteristics and the presence hybrid *Phoxinus* (DFA; df  = 68, Wilks' lambda  = 0.9507, p = 0.9192)(figure 2). We assume that our low estimated numbers of false absences in the subset of data used for the DFA analysis (1 for *P. neogaeus* and 2 for hybrids, based on a 9.4% rate of false absences) is unlikely to bias our results.

**Table 2:** Mean values of physiochemical variables for lakes with and without each *Phoxinus* species for the 70 lakes used in the discriminant function analysis.

	Group means and linear discriminant function coefficients for physiochemical variables							
	Area (ha)	Max. depth (m)	Elev. (m)	pH	Cond. (µS/cm)	DOC (ppm)	Ca (mg/L)	SO_4_ (mM)
*P. eos* present	196.54	22.21	392.62	6.43	36.02	4.27	3.19	7.66
absent	n/a	n/a	n/a	n/a	n/a	n/a	n/a	n/a
Hybrid present	183.82	24.18	391.71	6.46	35.45	4.18	3.13	7.62
absent	203.33	21.17	393.11	6.42	36.33	4.31	3.22	7.69
*coefficients*	*−0.002*	*0.058*	*−0.006*	*3.42*	*−0.113*	*0.232*	*−0.466*	*−0.013*
*P. neogaeus* present	239.07	16.60	322.78	6.70	41.44	4.64	3.73	7.81
absent	190.16	23.06	403.10	6.39	35.21	4.21	3.10	7.64
*coefficients*	*0.00002*	*−0.033*	*−0.017*	*1.86*	*−0.087*	*0.019*	*−0.194*	*0.227*

	Overall maximum and minimum values for physiochemical variables							
Maximum	2543.1	67.4	488	7.16	76	9.9	7.1	10
Minimum	3	1.5	165	5.77	23.7	1.5	1.94	3.57

Coefficients of the linear discriminant function (LD1) are provided only for *P. neogaeus* and hybrid *Phoxinus* because there were no lakes in the dataset without *P. eos*.

## Discussion

Hybrid *Phoxinus*, which are sperm-dependent asexuals, are widely distributed in Algonquin Park. The presence of hybrids in particular lakes was not correlated with the presence of a particular parental species. This observation suggests that the hybrids in Algonquin Park are generalists with respect to which parental species they use as a sperm donor. Also, the presence of hybrids was not correlated with the physiochemical characteristics of the lakes sampled in Algonquin Park. There was a significant negative correlation between the presences of the two parental species, *P. neogaeus* and *P. eos*, and there was a significant correlation between physiochemical lake characteristics and the presence of *P. neogaeus*. Assuming that these correlations are reflective of the known ecological differences (that are reflective of morphological differences) between *P. eos* and *P. neogaeus* [27], and that the distribution of *P. neogaeus* is limited by physiochemical factors, the lack of correlation between the presence of hybrids and physiochemical lake characteristics suggests that the hybrids in Algonquin Park are ecological generalists relative to the parental species.

Schlosser et al. [17] also found evidence that hybrid *Phoxinus* are generalists relative to the ecological niches of the parental species. The abundances of hybrid and parental *Phoxinus* from five adjacent drainages flowing into Lake Kabetogama along an 18 km stretch of shoreline in northern Minnesota suggested that all *Phoxinus* preferred the most oxygenated habitats [17]. The frequency of hybrids relative to the parental species was highest in habitats with lower oxygen concentration (i.e., more marginal habitats) [17]. The hybrids were also shown to survive longer than either parental species when held in very low or negligible oxygen concentrations [17] suggesting that the hybrids are ecological generalists relative to the parental species, at least in terms of tolerance for low oxygen concentration. Schlosser et al. [17] also found that the hybrids were intermediate between the two parental species in their trophic morphology. *Phoxinus eos* has a long and coiled intestine, a small mouth, a short narrow head, and a single row of slender pharyngeal teeth, while *P. neogaeus* has a short and relatively straight intestine, a large mouth, a long wide head, robust pharyngeal teeth, and a second row of two teeth on their pharyngeal jaw [13], [16], [17], [27], [28], [29]. Hybrids are essentially intermediate in all these morphological characters [13], [16], [17]. It has been shown, for the parental species at least, that this variation in trophic morphology is reflected in the diets of these fish – *P. eos* consumed more plant matter and algae and fewer macroinvertebrates than *P. neogaeus* [27]. No investigation of the diet of hybrid *Phoxinus* has been published, but it is reasonable to predict that, given their intermediate trophic morphology, the composition of the hybrids' diet would overlap with both parental species.

It should be noted that an implicit assumption in the present study is that there are no other lake characteristics (i.e., physiochemical characteristics not included in the analysis) that are relevant to *Phoxinus* distribution. It would be desirable to have a more complete data set with more lakes for which a larger suite of physiochemical variables has been measured. The present analysis is limited to eight abiotic variables, but an analysis with twice as many variables would also be limited to a certain degree. We must acknowledge, however, that we may have neglected a particular variable, or suite of variables, that differentiates the ecological niches of hybrid and parental *Phoxinus*. It is also possible that the range of values for the variables in the present analysis (i.e., within Algonquin Park) is small relative to the range throughout the distribution of *Phoxinus*. Important habitat differentiation may be affecting hybrid *Phoxinus* distribution at a larger scale.

There was a difference in the characteristics of the lakes that tended to be inhabited by the two parental *Phoxinus* species in Algonquin Park, and this difference may be reflective of the ecological differences between the parental species described above. It is possible, however, that the *P. neogaeus* distribution is the result of a lower dispersal ability relative to *P. eos*, or later arrival following post-glacial dispersal, both of which might explain why *P. neogaeus* is found primarily in lower elevation lakes with the physiochemical characteristics of such lakes. The fact that *P. neogaeus* is found in far fewer lakes than *P. eos* supports the hypothesis that their distribution is limited primarily by dispersal and colonization ability rather than by environmental factors. Dispersal and colonization ability may also account for the limited distribution of hybrids relative to *P. eos*. Hybrids are only able to colonize a lake after one of the parental species has arrived. Unlike *P. neogaeus*, however, there is no indication that the hybrids' ability to colonize a lake is limited by the physiochemical characteristics of that lake. An analysis of the combined influence on *Phoxinus* distribution of post-glacial dispersal distance and elevation would provide valuable insight into species coexistences in this system.

It is intriguing that no hybrid *Phoxinus* were found in lakes without *P. eos*, which may suggest that hybrid lineages in Algonquin Park are unable to solicit sperm from *P. neogaeus* males. There were, however, only 4 lakes with *P. neogaeus* that did not contain *P. eos*, which provides insufficient statistical power to draw inferences from the lack of hybrids in such cases. Also, hybrids were found in 9 lakes with both *P. neogaeus* and *P. eos*, and it remains unclear which parental species acts as the sperm-donor in those lakes. We are, therefore, limited in our ability to conclude that hybrid lineages in Algonquin Park are adapted to preferentially coexist with either parental species.

The reasons why sex is maintained as the dominant reproductive mode in vertebrates likely have to do with the rarity with which reversions to asexuality occur and with limits on the persistence of asexual lineages once they arise. In the present study, no limitations on the distribution of hybrid *Phoxinus* were detected. This may suggest that asexual reproduction is a successful strategy in this system (as opposed to most other vertebrate systems) because asexuals are able to survive and persist wherever their sperm donors are, regardless of the general physiochemical characteristics of their habitat, and likely regardless of which sperm donor is present. Further studies on the mechanisms allowing the persistence of both asexual and sexual *Phoxinus* across their range, especially concerning the ability of hybrids to solicit and successfully use sperm from the two parental species, are certainly warranted.

## References

[1] Agrawal AF (2006). Evolution of sex: why do organisms shuffle their genotypes?. Curr Biol.

[2] Dawley RM, Bogart JP (1989). Evolution and Ecology of Unisexual Vertebrates..

[3] Avise JC (2008). Clonality: The Genetics, Ecology, and Evolution of Sexual Abstinence in Vertebrates..

[4] Bell G (1982). The Masterpiece of Nature: The Evolution and Genetics of Sexuality..

[5] Darwin CR (1862). On the two forms, or dimorphic condition, in the species *Primula*, and on their remarkable sexual relations.. J Proc Linn Soc (Bot).

[6] Felsenstein J (1974). The evolutionary advantage of recombination.. Genetics.

[7] Maynard Smith J (1971). What use is sex?. J Theor Biol.

[8] Williams GC, Mitton JB (1973). Why reproduce sexually?. J Theor Biol.

[9] Schlupp I (2005). The evolutionary ecology of gynogenesis.. Annu Rev Ecol, Evol Syst.

[10] Goddard KA, Megwinoff O, Wessner LL, Giaimo F (1998). Confirmation of gynogenesis in *Phoxinus eos-neogaeus* (Pisces: Cyprinidae).. J Hered.

[11] Mee JA, Rowe L (2006). A comparison of parasite loads on asexual and sexual *Phoxinus* (Pisces: Cyprinidae).. Can J Zool-Rev Can Zool.

[12] Mee JA, Otto SP (2010). Variation in the strength of male mate choice allows long-term coexistence of sperm-dependent asexuals and their sexual hosts..

[13] New JG (1962). Hybridization between two cyprinids, *Chrosomus eos* and *Chrosomus neogaeus*.. Copeia.

[14] Angers B, Schlosser IJ (2007). The origin of *Phoxinus eos-neogaeus* unisexual hybrids.. Mol Ecol.

[15] Dawley RM, Schultz RJ, Goddard KA (1987). Clonal reproduction and polyploidy in unisexual hybrids of *Phoxinus eos* and *Phoxinus neogaeus* (Pisces; Cyprinidae).. Copeia.

[16] Goddard KA, Dawley RM, Dowling TE, Dawley RM, Bogart JP (1989). Origin and genetic relationships of diploid, triploid, and diploid-triploid mosaic biotypes in the *Phoxinus eos-neogaeus* unisexual complex.. Evolution and Ecology of Unisexual Vertebrates.

[17] Schlosser IJ, Doeringsfeld MR, Elder JF, Arzayus LF (1998). Niche relationships of clonal and sexual fish in a heterogeneous landscape.. Ecology.

[18] Elder JF, Schlosser IJ (1995). Extreme clonal uniformity of *Phoxinus eos/neogaeus* gynogens (Pisces: Cyprinidae) among variable habitats in northern Minesota beaver ponds.. Proc Natl Acad Sci U S A.

[19] Binet MC, Angers B (2005). Genetic identification of members of the *Phoxinus eos-neogaeus* hybrid complex.. J Fish Biol.

[20] Doeringsfeld MR, Schlosser IJ, Elder JF, Evenson DP (2004). Phenotypic consequences of genetic variation in a gynogenetic complex of *Phoxinus eos-neogaeus* clonal fish (Pisces: Cyprinidae) inhabiting a heterogeneous environment.. Evolution.

[21] Massicotte R, Magnan P, Angers B (2008). Intralacustrine site fidelity and nonrandom mating in the littoral-spawning northern redbelly dace (Phoxinus eos).. Can J Fish Aquat Sci.

[22] Quinlan R, Paterson AM, Hall RI, Dillon PJ, Wilkinson AN (2003). A landscape approach to examining spatial patterns of limnological variables and long-term environmental change in a southern Canadian lake district.. Freshwat Biol.

[23] R Development Core Team (2010). R: A Language and Environment for Statistical Computing.. http://www.R-project.org.

[24] Cohen J (1960). A coefficient of agreement for nominal scales.. Educ Psych Meas.

[25] Cohen J (1968). Weighted kappa: nominal scale agreement with provisions for scaled disagreement or partial credit.. Psychol Bull.

[26] Titus K, Mosher JA, Williams BK (1984). Chance-corrected classification for use in discriminant analysis - ecological applications.. Am Midl Nat.

[27] Cochran PA, Lodge DM, Hodgson JR, Knapik PG (1988). Diets of syntopic finescale cace, *Phoxinus neogaeus*, and northern redbelly dace, *Phoxinus eos* - a reflection of trophic morphology.. Environ Biol Fishes.

[28] Phillips GL (1969). Morphology and variation of american cyprinid fishes *Chrosomus erythrogaster* and *Chrosomus eos*.. Copeia.

[29] Stasiak RH (1977). Morphology and variation in finescale dace, *Chrosomus neogaeus*.. Copeia.

